# Role of intraretinal cysts in the prediction of postoperative closure and photoreceptor damages of the idiopathic full-thickness macular hole

**DOI:** 10.1186/s12886-021-02204-x

**Published:** 2022-01-03

**Authors:** Jin-Ho Joo, Woo Ho Nam, Taesung Joo, Sang Woong Moon

**Affiliations:** 1 Department of Ophthalmology, Kyung Hee University Hospital at Gangdong, Gangdong, 892 Dongnam-ro, Gangdong-gu, Seoul, 05278 Republic of Korea; 2 Division of Ophthalmology, Department of Medicine, Kyung Hee University Graduate School, Seoul, Republic of Korea

**Keywords:** Macular hole, Intraretinal cyst, Hole closure, Photoreceptor damages, Macular hole closing factor

## Abstract

**Background:**

To determine whether it would be effective in predicting the results of the postoperative full-thickness macular hole (FTMH) closure when intraretinal cyst (IRC) is present.

**Methods:**

Case-control study. Patients with idiopathic FTMH who underwent pars plana vitrectomy with internal limiting membrane peeling were retrospectively reviewed. Preoperative spectral-domain optical coherence tomography was undertaken in all patients. The new parameter, macular hole closing factor (MHCF) was defined as the base diameter - (arm length + IRC height) by adding IRC to the existing parameter. After surgery, patients were classified and analyzed according to the type of hole closure and the damage of photoreceptor.

**Results:**

Of the 35 patients, 28 (80.00%) had type 1 closure and seven (20.00%) had type 2 closure. There was a significant difference in postoperative BCVA (*P* < 0.01), base diameter (*P* = 0.037), arm length (*P* = 0.045), and IRC height (*P* = 0.011) between the two groups. In the type 1 closure, they were further divided into two subgroups according to photoreceptor damage, and it was confirmed that there were significant differences in postoperative BCVA (*P* = 0.045), hole height (*P* = 0.048), and IRC height (*P* = 0.046) in the two subgroups. As for the new parameters, a significant difference between the three groups was confirmed (*P* < 0.01).

**Conclusion:**

IRC may help predict hole closure along with the known horizontal parameters. Therefore, the new parameter containing both two factors can help predict not only hole closure but also damage to photoreceptors that affects postoperative visual prognosis.

**Supplementary Information:**

The online version contains supplementary material available at 10.1186/s12886-021-02204-x.

## Background

Idiopathic full-thickness macular hole (FTMH) is a disease that shows cystic changes accompanied by a defect in the neurosensory retina at the macula and is accompanied by severe central vision loss. The pathogenesis was reported to be due to anteroposterior and tangential traction forces by the posterior vitreous membrane and internal limiting membrane (ILM), respectively [1, 2]. With the advent of spectral-domain (SD) optical coherence tomography (OCT) is a useful diagnostic tool for the diagnosis and observation of FTMH. It can accurately measure the height of the hole and hole distance [3–5]. The success rate of FTMH after vitrectomy was 69–80.6% [6–8]. Nevertheless, anatomical success cannot be achieved in all FTMH cases [9]. Therefore, several studies have attempted to confirm the closure of FTMHs through OCT.

Among OCT parameters, studies have determined the anatomic success of MH surgery according to the size of the hole diameter [10, 11]. Among them, the base diameter was found to be the most useful parameter [12]. In addition to hole size, various studies have been published explaining derivative indicators; macular hole index (MHI), tractional hole index, hole form factor (HFF), and diameter hole index [11, 13, 14]. The disadvantage with these indices is that they consider primarily one of the two tractional forces for MH formation. The MH size and its elevated edematous edges on clinical examination primarily occur depending on the extent of anteroposterior and tangential tractional forces acting on the hole. Intraretinal fluid and retinal tissue can be important determining factors in predicting the likelihood of surgical success by allowing them to determine the extent of tangential and anteroposterior tractional forces. Another study has confirmed that the cystoid space area is an excellent predictor of MH closure [15]. Another study showed better anatomic closure and improvement of best-corrected visual acuity (BCVA) in the MH configuration with a cuff of subretinal fluid at the broken margin [16].

Although hole closure is important, it is also important to restore BCVA after FTMH surgery. It was found that there is a strong correlation with postoperative BCVA depending on the presence of photoreceptor inner/outer segment defect in SD-OCT after surgery [17]. Another study found that recovery of the external limiting membrane (ELM) and ellipsoid zone (EZ) line at fovea correlated with postoperative BCVA [18].

Few previous studies have reported that the presence of intraretinal cyst (IRC) is associated with the successful closure of FTMH. In the case of traumatic MH, it has been reported that the presence of IRC may predict spontaneous closure [19]. There was a report that the closure rate of the hole could be increased if CME was treated with topical therapy in secondary FTMH [20]. Moreover, only a few studies have shown that it is associated with preoperative BCVA [21] and metamorphopsia [22]. Although the presence of IRC is assumed to have a significant effect on the morphological changes of MH, there have been no studies related to postoperative MH closure.

Therefore, this study aimed to determine whether the presence of IRC before surgery in patients with FTMH is correlated with postoperative FTMH closure and photoreceptor damage.

## Methods

### Subjects

This retrospective and case-control study involved patients with idiopathic FTMH who visited the Department of Ophthalmology at Kyung Hee University Hospital at Gangdong and underwent MH surgery from March 2016 to July 2020. The current research followed the tenets of the Declaration of Helsinki, and all patients provided informed consent after an explanation of the study protocol. The Institutional Review Board at Kyung Hee University Hospital at Gangdong (KHNMC-2021-02-016) approved this retrospective study. Formal consent for publication was obtained from relevant patient. The following inclusion criteria were used: (1) pars plana vitrectomy with ILM peeling using 23 G vitrectomy system and indocyanine green dye to stain the ILM by a single surgeon (S.W.M) and (2) a minimum follow-up period of 6 months. Exclusion criteria included the following: (1) secondary MH such as trauma, high myopia, postsurgical, and diabetic macular edema; (2) history of previous vitreoretinal surgery; (3) history of any treatment related to retinal disease; and (4) any other ocular pathology that could contribute to visual loss.

### Ocular examinations

All patients underwent a complete ophthalmologic examination, including an assessment of BCVA, slit-lamp examination, pupil-dilated fundus examination, color fundus photography, and OCT. Snellen BCVA was recorded before and 6 months after surgery and was converted to Logarithm of the Minimum Angle of Resolution (logMAR) BCVA for analysis. The OCT images were obtained using SD-OCT (Spectralis; Heidelberg Engineering, Heidelberg, Germany) before and 6 months after surgery.

### Measurements of SD-OCT parameters

**Fig. 1:**
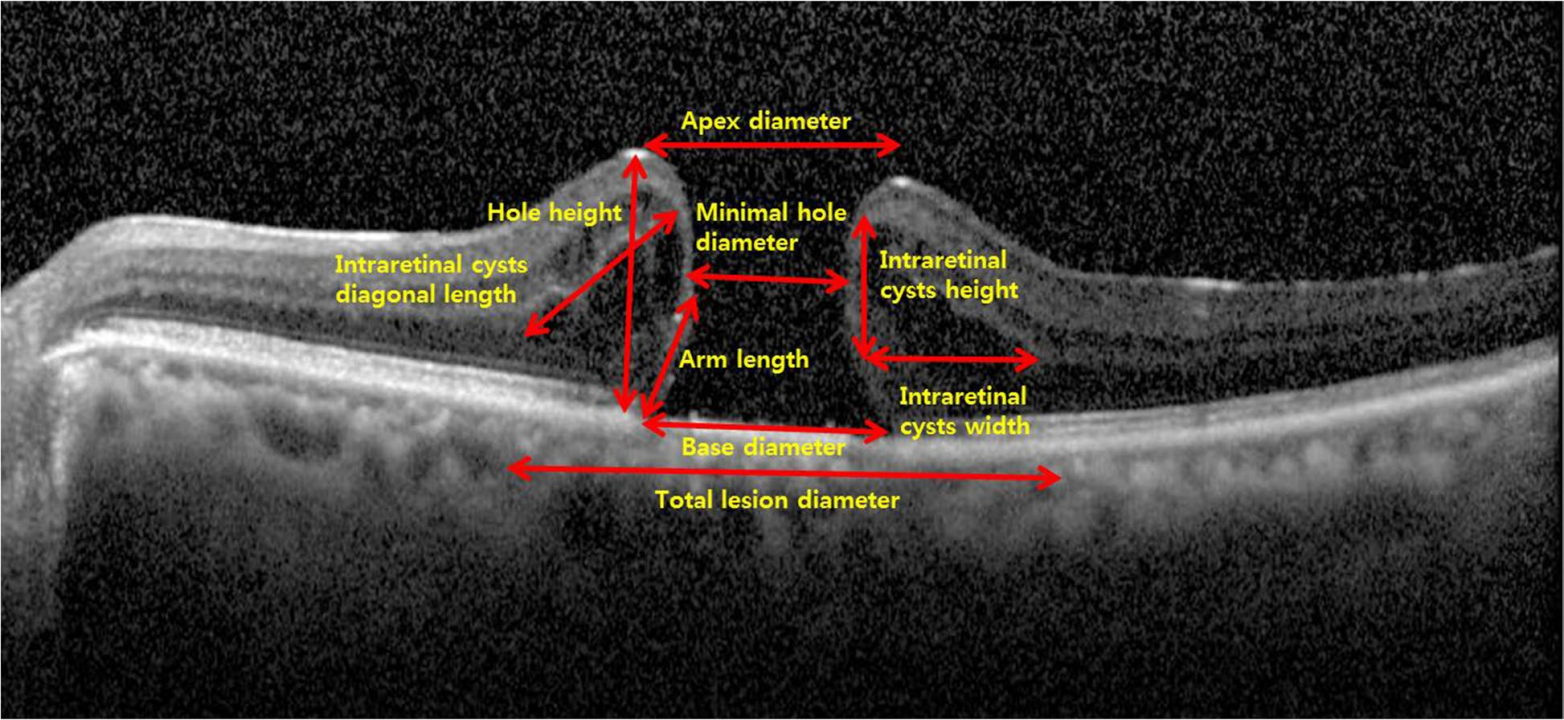
Preoperative parameters of macular hole on spectral domain optical coherence tomography

The preoperative SD-OCT image was analyzed by four observers (J.H.J, W.H.N, T.J, and S.W.M) using Spectralis viewing module calculator manager (version 1.0.15.0, Heidelberg Engineering, Heidelberg, Germany). Using the measurement system in the viewer, the following figures were measured as in Fig. 1; base, minimal hole, apex, and total lesion diameter; arm length; hole height; and IRC width, height, and diagonal length. The arm and IRC angle were measured using the Image J image analysis software (version 1.52, Wayne Rasband, National Institutes of Health, Bethesda, MD, USA). In the SD-OCT image, the diameter value was averaged by measuring the values of the horizontal and vertical planes, and the parameters of hole height, arm, and IRC were recorded by averaging values in both directions in the horizontal (temporal and nasal sides) and vertical (superior and inferior sides) planes at the center of the MH. We measured a new parameter, macular hole closing factor (MHCF) to ensure that the pulled retinal plane could sufficiently cover the hole after the traction of the membrane was released after surgery. It was the value of base diameter minus the sum of arm length and IRC height.

### Anatomical outcomes

In our study, successful closure of FTMH was defined as the disappearance of the rim of the MH or attachment to the underlying retinal pigment epithelium (RPE), with the flattening of the cuff of retinal detachment around the hole after the operation. Based on the postoperative SD-OCT findings, we classified FTMH into two groups; type 1 and type 2 as defined by Kang et al. [23]. Type 1 closure was designated when MH was closed without foveal defect of the neurosensory retina, and type 2 closure occurred when a foveal defect of the neurosensory retina persisted postoperatively; although, the whole rim of the MH was attached to the underlying RPE with flattening of the cuff (bare RPE). We confirmed the defect of the photoreceptor using SD-OCT in the type 1 group; thus, the intact and damaged foveal photoreceptor groups were further classified. The postoperative damaged foveal photoreceptor group was defined as the case of ELM, or/and EZ line damages in SD-OCT (Supplementary Fig. 1). The average was obtained by measuring the horizontal and vertical lengths of the damaged part of the photoreceptor, and if both the ELM and EZ lines were damaged, the damaged part was determined as a larger part.

### Statistical methods

Statistical analysis was performed using the Statistical Package for the Social Sciences software 25.0 (IBM, Armonk, New York, USA). Data were compared using the Mann-Whitney test and Fishers exact test. The statistical significance of the BCVA change following surgery was confirmed through the paired t-test and Wilcoxon test. A receiver operating characteristic (ROC) curve was constructed to obtain cutoff values of SD-OCT parameters for the prediction of MH closure and damages of the foveal photoreceptor.

## Results

In total, 35 eyes of 35 patients were enrolled (12 men [34.29%] and 23 women [65.71%]). The average age of the participants was 69.3 ± 7.0 years (range: 53–90) years. Of 35 eyes, 24 (68.57%) patients were in phakic states and underwent combined cataract surgery, and later presented with pseudophakia. The remaining 11 (31.43%) were in pseudophakic states at the time of surgery. All 35 eyes underwent ILM peeling and gas tamponade. The mean preoperative BCVA (logMAR) value was 0.93 ± 0.33, and the mean postoperative BCVA (logMAR) value 6 months after surgery was 0.59 ± 0.41. There was a significant improvement in BCVA values (paired t-test, *p* < 0.01). The mean time to surgery due to the onset of symptoms was 8.5 ± 10.4 weeks. The mean base diameter was 887.0 ± 276.3 μm.

### Comparison between postoperative type 1 and type 2 closed full-thickness macular hole groups

Of the 35 FTMH patients in this study, 28 (80.00%) and seven (20.00%) were diagnosed as type 1 and 2 closed FTMH, respectively. In the Type 1 closed FTMH group of 28 patients, vitreomacular traction (VMT) was observed in 9 patients (32.14%) and epiretinal membrane (ERM) in 11 patients (39.29%). In the type 2 closed FTMH group of 7 patients, VMT was not observed, and ERM was observed in 2 patients (28.57%). It was not confirmed that VMT and ERM had a statistically significant effect on the type of MH closure The mean preoperative BCVA (logMAR) values were 0.88 ± 0.30 in the type 1 closed FTMH group and 1.09 ± 0.40 in the type 2 closed FTMH group, and there was no statistically significant difference between the two groups (*p* = 0.294). However, the mean postoperative BCVA (logMAR) values in type 1 and 2 closed FTMH groups were 0.46 ± 0.24 and 1.11 ± 0.45, respectively. A statistically significant difference was noted in the postoperative BCVA value between the two groups (*p* < 0.01). After operating, there was a significant improvement in BCVA values in type 1 closed FTMH group (Wilcoxon test, p < 0.01); however, statistical significance was not confirmed in type 2 closed FTMH group (Wilcoxon test, *p* = 0.680) (Table 1).

**Table 1:** Comparison of demographics and the average value of spectral-domain optical coherence tomography parameters between postoperative type 1 between type 2 closed full-thickness macular hole groups

	Postoperative Type 1 Closed FTMH (*n* = 28)	Postoperative Type 2 Closed FTMH (*n* = 7)	*p* value*
Age (years)	68.0 ± 5.6	74.3 ± 9.9	0.139
Sex (male: female)	9:19	3:4	
Duration (weeks)	7.4 ± 8.1	12.7 ± 16.8	0.357
Combined cataract operation (%)	18 (64.29%)	6 (85.71%)	0.621
Vitreomacular traction (%)	9 (32.14%)	0 (0%)	0.151^†^
Epiretinal membrane (%)	11 (39.29%)	2 (28.57%)	0.682^†^
Best-corrected visual acuity (logMAR)			
Preoperative	0.88 ± 0.30	1.09 ± 0.40	0.294
Postoperative	0.46 ± 0.24	1.11 ± 0.45	**< 0.01**
	**< 0.01**^**††**^	0.680^**††**^	
Base diameter (μm)	837.0 [643.0, 1006.0]	1030.0 [780.5, 1134.5]	**0.037**
Minimal hole diameter (μm)	285.0 [210.0, 491.5]	532.0 [360.0, 638.0]	**0.015**
Apex diameter (μm)	785.0 [635.0, 992.5]	1063.0 [911.0, 1239.0]	**< 0.01**
Total lesion diameter (μm)	2835.8 [2468.4, 3313.6]	2856.0 [2498.0, 3033.5]	0.880
Arm length (μm)	353.5 [241.3, 438.3]	287.3 [220.8, 314.8]	**0.045**
Arm angle	37.7 [35.2, 45.8]	40.9 [35.2, 44.2]	0.836
Hole height (μm)	434.0 [382.8, 486.5]	366.0 [323.8, 448.0]	0.127
IRC width (μm)	728.5 [607.0, 949.0]	657.8 [567.0, 865.8]	0.531
IRC height (μm)	232.0 [176.3, 274.5]	132.3 [117.0, 202.3]	**0.011**
IRC diagonal length (μm)	621.5 [515.0, 804.5]	507.8 [356.0, 668.0]	0.294
IRC angle	17.3 [13.8, 20.6]	16.4 [13.1, 19.7]	0.573
Hole form factor (HFF)	0.83 [0.69, 0.93]	0.55 [0.51, 0.57]	**< 0.01**
Macular hole index (MHI)	1.06 [0.85, 1.15]	0.79 [0.59, 1.13]	0.072
Base diameter – (arm length + IRC height) (μm)	−246.5 [− 375.0, − 148.0]	48.5 [− 16.5, 191.0]	**< 0.01**

Macular hole parameters are expressed as median (interquartile range)

FTMH: full thickness macular hole; IRC: Intraretinal cysts; Hole form factor: (nasal arm length + temporal arm length) / base diameter; Macular hole index: Hole height / base diameter

Boldface indicate *p* < 0.05

* Comparative analysis of two groups using Mann-Whitney test

† Fishers exact test was used to determine whether it affects the type of macular hole closure

†† Statistical significance of visual acuity changes through Wilcoxon test

Among the measured SD-OCT parameters, there were statistically significant differences between base diameter (*p* = 0.037), minimal hole diameter (*p* = 0.015), apex diameter (*p* < 0.01), arm length (*p* = 0.045), IRC height (*p* = 0.011), and HFF (p < 0.01). As for the MHCF, a statistically significant difference between the two groups was confirmed (*p* < 0.01) (Table 1).

### Subgroup analysis according to the postoperative damage of foveal receptor

We subgrouped type 1 closed FTMH group additionally according to the postoperative damage of foveal photoreceptor observed on SD-OCT (Table 2). Thirteen (46.43%) of 28 eyes had intact foveal photoreceptor closure; however, 15 (53.57%) had damaged foveal photoreceptor closure. There was a statistically significant difference in postoperative BCVA (0.35 ± 0.24 vs. 0.56 ± 0.27, p = 0.045). The improvement of BCVA after surgery was significantly improved in both groups (Wilcoxon test, *p* < 0.01). In the damaged foveal photoreceptor closure group, the average diameter of the damaged photoreceptor diameter was 354.6 ± 440.8 μm. It was confirmed that there was a statistically significant between the two groups in hole height (*p* = 0.048), IRC height (*p* = 0.046) and HFF (*p* = 0.031). As for the MHCF, a statistically significant difference between the two groups was confirmed (*p* < 0.01).

**Table 2:** Comparison of the average value of two sub-groups according to the damage of foveal photoreceptor by using spectral-domain optical coherence tomography parameters in type 1 closed full-thickness macular hole patients

	Intact foveal photoreceptor closure (*n* = 13)	Damaged foveal photoreceptor closure (*n* = 15)	p value*
Best corrected visual acuity (logMAR)			
Preoperative	0.78 ± 0.29	0.97 ± 0.29	0.116
Postoperative	0.35 ± 0.24	0.56 ± 0.27	**0.045**
	**< 0.01**^**†**^	**< 0.01**^**†**^	
Durations (week)	8.9 ± 9.4	6.1 ± 6.7	0.375
Defected photoreceptor diameter (μm)	–	354.6 ± 440.8	
Base diameter (μm)	837.0 [705.5, 1080.8]	813.8 [609.9, 1047.3]	0.943
Minimal hole diameter (μm)	292.5 [181.3, 517.8]	282.0 [237.7, 464.8]	0.650
Apex diameter (μm)	800.0 [775.3, 922.3]	671.3 [476.6, 1017.4]	0.169
Total lesion diameter (μm)	2747.0 [2477.1, 2961.5]	2954.8 [2344.5, 3457.8]	0.527
Arm length (μm)	395.8 [250.9, 456.8]	329.1 [235.8, 384.5]	0.202
Arm angle	38.5 [35.5, 45.6]	36.2 [35.0, 48.9]	0.924
Hole height (μm)	443.3 [407.9, 518.6]	414.8 [374.9, 452.9]	**0.048**
IRC width (μm)	758.0 [618.8, 896.8]	726.5 [469.2, 958.2]	0.430
IRC height (μm)	240.0 [204.6, 294.9]	196.4 [168.0, 247.1]	**0.046**
IRC diagonal length (μm)	630.0 [543.0, 820.4]	620.4 [455.9, 805.9]	0.583
IRC angle	19.7 [15.8, 23.4]	14.8 [13.8, 17.5]	0.175
Hole form factor (HFF)	0.92 [0.75, 0.97]	0.70 [0.67, 0.87]	**0.031**
Macular hole index (MHI)	1.08 [0.94, 1.15]	0.92 [0.84, 1.30]	0.503
Base diameter – (arm length + IRC height) (μm)	−375.0 [− 465.0, −202.0]	− 192.5 [− 304.0, −93.3]	**< 0.01**

Macular hole parameters are expressed as median (interquartile range)

FTMH: full thickness macular hole; IRC: Intraretinal cysts; Hole form factor: (nasal arm length + temporal arm length) / base diameter; Macular hole index: Hole height / base diameter

Boldface indicate p < 0.05

* Comparative analysis of two groups using Mann-Whitney test

† Statistical significance of visual acuity changes through Wilcoxon test

**Fig. 2:**
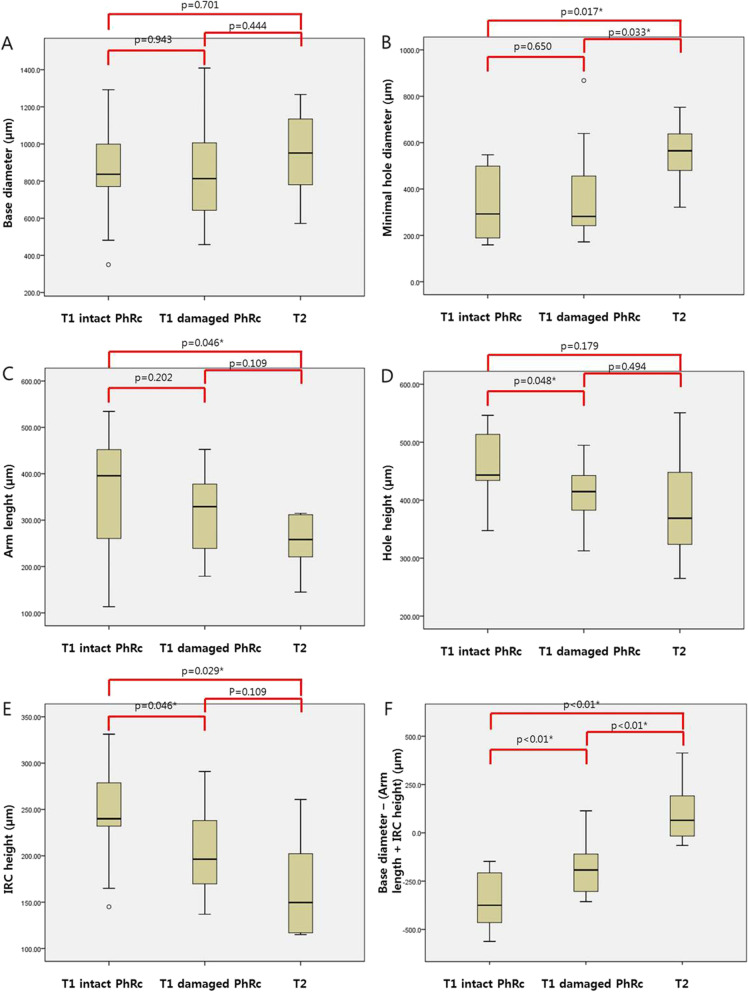
Graph showing difference of parameters of spectral domain optical coherence tomography for 3 sub-group; intact foveal photoreceptor type 1 closure (n = 13), damaged foveal photoreceptor type 1 closure (*n* = 14), and type 2 closure (n = 7). The Mann-Whitney test was used, and *p* values less than 0.05 were marked with an asterisk (*). (T1: type 1 closure, T2: type 2 closure, PhRc: photoreceptor)

The Mann-Whitney test was used to compare the three groups; intact foveal photoreceptor type 1 closure, damaged foveal photoreceptor type 1 closure, and type 2 closure FTMH. The results are shown in Fig. 2. It was confirmed that there was a difference in hole height (p = 0.048), IRC height (p = 0.046), and new parameter (p < 0.01) between the intact and the damaged foveal photoreceptor group of the type 1 closure group. Between intact foveal photoreceptor type 1 and 2 closure groups, it was confirmed that there were statistically significant differences in minimal hole diameter (*p* = 0.017), arm length (p = 0.046), IRC height (*p* = 0.029), and the MHCF (p < 0.01). Between damaged foveal photoreceptor type 1 and 2 closure groups, it was confirmed that there were statistically significant differences in minimal hole diameter (*p* = 0.033), and the MHCF (p < 0.01).

### Receiver operating characteristic (ROC) curves as predictors of FTMH closure and intact foveal photoreceptor

**Fig. 3:**
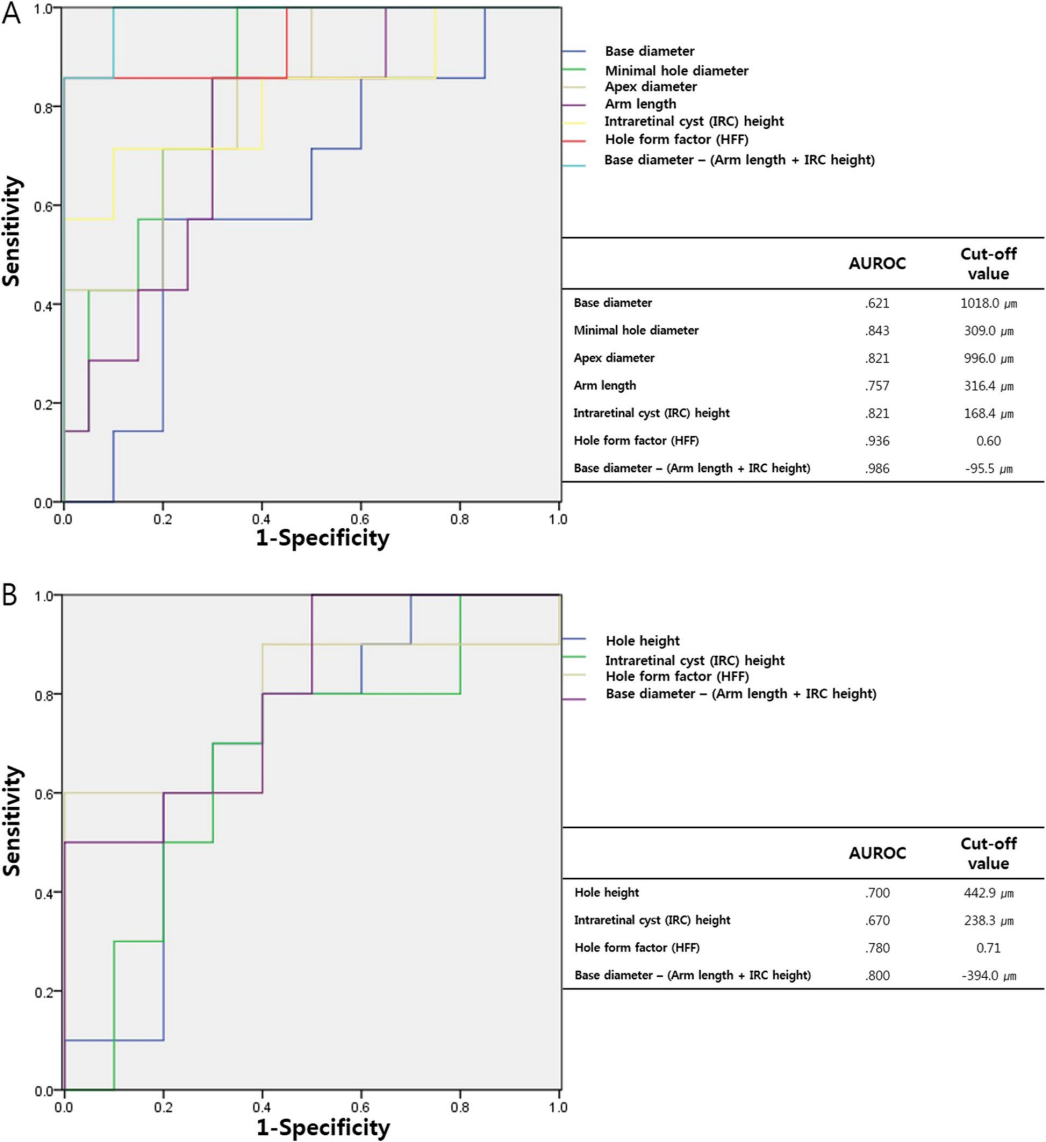
Receiver operating characteristic (ROC) curves obtained for parameters of spectral domain optical coherence tomography as prognostic factor for distinguishing the closure type of FTMH (**A**) and whether the photoreceptor is intact in type 1 closed FTMH (**B**)

Figure 3 shows the ROC curve for the parameters of SD-OCT used as a prognostic factor for distinguishing the closure type of FTMH and assessing whether the foveal photoreceptor is intact in postoperative type 1 closed FTMH.

In the ROC curve to help to distinguishing the closure type of FTMH, the area under the ROC curve (AUROC) for the base diameter is 0.621, minimal hole diameter is 0.843, apex diameter is 0.812, arm length is 0.757, IRC height is 0.821, HFF is 0.936 and the MHCF is 0.986. The cutoff value for distinguishing the closure type of FTMH is 1018.0 μm (sensitivity: 57.1%, specificity: 80.0%) in the base diameter, 309.0 μm (sensitivity: 100.0%, specificity: 65.0%) in the minimal hole diameter, 996.0 μm (sensitivity: 71.4%, specificity: 80.0%) in the apex diameter, 316.4 μm (sensitivity: 85.7%, specificity: 70.0%) in the arm length, 168.4 μm (sensitivity: 71.4%, specificity: 90.0%) in the IRC height, 0.60 (sensitivity: 85.7%, specificity: 100.0%) in the HFF, and − 95.50 μm (sensitivity: 100.0%, specificity: 90.0%) in the MHCF.

In the ROC curve to help to to predict whether the photoreceptor is intact in type 1 closed FTMH, the AUROC for the hole height is 0.700, IRC height is 0.670, HFF is 0.780 and the MHCF is 0.800. The cutoff value for predicting the photoreceptor intact in type 1 closed FTMH is 442.9 μm (sensitivity: 80.0%, specificity: 60.0%) in the hole height, 238.3 μm (sensitivity: 80.0%, specificity: 60.0%) in the IRC height, 0.71 (sensitivity: 60.0%, specificity: 100.0%) in the HFF and − 394.0 μm (sensitivity: 100.0%, specificity: 50.0%) in the MHCF.

## Discussion

The results of our study can be summarized as follows: (1) It could help predict the closure of FTMH through SD-OCT parameters: base diameter, minimal hole diameter, apex diameter, arm length, IRC height, MHCF: base diameter - (arm length + IRC height). (2) It may be helpful to use SD-OCT parameters to predict photoreceptor damage in type 1 FTMH closure group; hole height, IRC height, MHCF (3) In idiopathic FTMH, the presence of IRC could help predict hole closure as well as foveal photoreceptor damage.

MH surgery aims to remove the traction force of the vitreous body and ILM pulling the retina, close the hole, and detach the contraction-inducing tissue around the fovea. To obtain an additional tamponade effect, the gas-liquid exchange is performed to promote the hole closure. The success or failure of the surgery can be divided into anatomical success, which indicates recovery to the normal retinal shape; and functional success, which indicates more than two lines of vision improvement; however, visual acuity outcomes are not always satisfactory even with anatomical success [24].

First, in this study, as in previous studies, idiopathic FTMH showed statistically significant improvement in visual acuity after type 1 closure, and postoperative BCVA was better than type 2 closure [23]. In previous studies, type 1 closure tended to be compared more to type 2 when the MH diameter was smaller [15, 23]. In our study, it was confirmed that some horizontal parameters; base diameter, minimal hole diameter, and apex diameter were smaller in type 1 closure group than in type 2 closure. Additionally, it was confirmed that the measured arm length and IRC height values were more significant in type 1 closure group than in type 2 group. In other studies, the larger HFF, the value obtained by dividing the arm length by the maximum basal diameter, is helpful for MH closure [11, 15]. Therefore, a large arm length will affect the successful anatomic success. In the case of IRC height, there was no direct comparison in previous studies; however, according to Helmut [16], there was a higher probability of hole closure in the presence of cuff of subretinal fluid on OCT. Furthermore, the probability of anatomical success increases with increase in the number of cystic spaces in MH [15]. Therefore, we confirmed that the horizontal parameters help the successful closure of the FTMH as in the previous study, and the vertical parameters arm length and IRC height are important parameters for FTMH closure.

Second, our researchers further divided groups according to the presence or absence of foveal photoreceptor damage in type 1 closure group. The degree of improvement was found to be better when the photoreceptor was intact. Similar to previous studies, it was confirmed that if there is no photoreceptor damage, the postoperative visual acuity results tend to be remarkable [17, 18]. Comparing the intact and damaged photoreceptor groups, the values of the hole and IRC height among the SD-OCT parameters showed a larger average value in the intact photoreceptor group, with a significant difference.

Third, when we compared the three groups; intact photoreceptor type 1 closure group, damaged photoreceptor type 1 closure group, and type 2 closure group, it was confirmed that the MHCF had a significant difference in the 3 groups. It can be assumed that horizontal parameters mainly affect the distinction between type 1 and type 2 closure, and vertical parameters influence the prediction of photoreceptor damage in the type 1 closure group (Table 2, Fig. 3). The MHCF was introduced in this study owing to the assumption that the base diameter was the area to be restored or that when the nerve tissues were missing, the arm length and IRC height could represent the amount of nerve tissues that could fill the defect area. Therefore, it could predict the anatomical success of FTMH better than the conventional HFF or MHI (hole height divided by basal diameter) as the advantage of this parameter includes both horizontal and vertical factors.

It was confirmed that the type of MH closure had no effect on the presence of VMT or ERM before FTMH surgery (Table 1). VMT has long been known to affect the formation of MH [3]. However, in previous studies, it is known that the presence of VMT does not affect successful closure after operation in FTMH [25]. In this study, it was confirmed that the presence of VMT had no effect on FTMH closure. It has been reported that the presence of ERM is associated to the prognosis of FTMH closure, but the same result could not be confirmed in this study due to the insufficient number of samples [26].

There was a study analyzing the correlation between the area of IRC and hole closure measured using Image J software in patients with MH [15]. It is assumed that measuring the IRC height is more direct and convenient than measuring the IRC area. Additionally, our study was analyzed three-dimensionally as parameters were measured not only on the nasal and temporal side of the horizontal plane but also on the superior and inferior side of the vertical plane.

Since cystic changes in the neural retinal layer affect the visual acuity negatively, if it is assumed that the height of the hole edge is due to cystic changes; thus, the increase in height can presumably worsen the visual acuity. However, the results of this study were converse, that is, the surgical outcome was improved as the size of the cyst increased. Therefore, cystic change is considered to be an indicator of functional retinal tissue. The cystic change is assumed with good visual acuity after surgery as significant functional retinal tissues persist. Moreover, if the MH height is lowered due to atrophy of the neural retinal tissues, it is presumed to be related to the failure of the operation or less recovery of the visual acuity after surgery. The success of surgery or postoperative visual acuity is better when the hole diameter is smaller in size. The base diameter is the area to be restored or where the nerve tissue is damaged, and the height of the IRC or the length of the arm indicates the amount of nerve tissue that can fill the defect area; therefore, it will help predict the success of the surgery.

According to previous studies, when the failed to close after first surgery or reopened, reoperation is considered necessary from the point of view of recovery of visual acuity. It is known that the results of reoperation are anatomically successful in 78–85% of cases. However, it is necessary to discuss the selection criteria for the revision surgery to obtain better surgical results [27, 28]. Our research team confirmed that the increase in IRC height increases the success rate of reoperation through the several cases (Supplementary Fig. 2) Through this, we consider that the increase in IRC height will have a positive effect on primary surgery as well as revision of FTMH.

In this study, the duration of symptom onset in type 1 and type 2 closure were 7.4 ± 8.1 weeks and 12.7 ± 16.8 weeks, respectively; however, there was no significant difference due to the large deviation. In previous studies, the anatomical success rate was high in cases less than 6 months after the onset of symptoms, [29] and the eyesight improvement effect was high in cases less than 2 months [30]. It is challenging to accurately predict the time of symptom onset due to MH as the period is not consistent with the MH period, and is likely to be confused with the symptoms of other ophthalmic diseases. And, the mean base diameter was 887.0 ± 276.3 μm in this study. Since the MH size of the operated patients was relatively large, it is thought that 80% of type 1 closures were recorded.

The limitations of this study include a relatively small sample size and a short follow-up period. Further studies with a larger sample and longer follow-up period will be needed. If the sample size is larger, the correlation between the above parameters and the degree of visual acuity or photoreceptor damage must be analyzed. In addition, IRC is considered to be a factor that can determine reoperation in case of failure of the first MH operation, so a large-scale study is needed.

## Conclusions

In conclusion, in FTMH, IRC may help predict not only hole closure but also damage to photoreceptors that affects postoperative visual prognosis along with the previously widely known horizontal parameters. And we believe that when the first operation fails, waiting for IRC to develop and then reoperation will have better results.

## Supplementary Information

**Additional file 1.**

## Data Availability

Not applicable.

## Abbreviations

FTMH: full-thickness macular hole
IRC: intraretinal cyst
ILM: internal limiting membrane
SD: spectral-domain
OCT: optical coherence tomography
MHI: macular hole index
HFF: tractional hole index, hole form factor
BCVA: best-corrected visual acuity
ELM: external limiting membrane
EZ: ellipsoid zone
logMAR: Logarithm of the Minimum Angle of Resolution
MHCF: macular hole closing factor
RPE: retinal pigment epithelium
ROC: receiver operating characteristic
VMT: vitreomacular traction
ERM: epiretinal membrane

## References

[1] Gass JD (1988). Idiopathic senile macular hole. Its early stages and pathogenesis. Archives of ophthalmology (Chicago, Ill : 1960).

[2] Smiddy WE, Flynn HW (2004). Pathogenesis of macular holes and therapeutic implications. Am J Ophthalmol.

[3] Duker JS, Kaiser PK, Binder S, de Smet MD, Gaudric A, Reichel E, Sadda SR, Sebag J, Spaide RF, Stalmans P (2013). The international Vitreomacular traction study group classification of vitreomacular adhesion, traction, and macular hole. Ophthalmology.

[4] Uchino E, Uemura A, Ohba N (2001). Initial stages of posterior vitreous detachment in healthy eyes of older persons evaluated by optical coherence tomography. Archives of Ophthalmology (Chicago, Ill : 1960).

[5] Koizumi H, Spaide RF, Fisher YL, Freund KB, Klancnik JM, Yannuzzi LA (2008). Three-dimensional evaluation of vitreomacular traction and epiretinal membrane using spectral-domain optical coherence tomography. Am J Ophthalmol.

[6] Freeman WR, Azen SP, Kim JW, el-Haig W, Mishell DR, 3rd, Bailey I: Vitrectomy for the treatment of full-thickness stage 3 or 4 macular holes. Results of a multicentered randomized clinical trial. The Vitrectomy for Treatment of Macular Hole Study Group. Archives of Ophthalmology (Chicago, Ill : 1960) 1997, 115(1):11–21. 10.1001/archopht.1997.01100150013002 9006420

[7] Ezra E, Gregor ZJ: Surgery for idiopathic full-thickness macular hole: two-year results of a randomized clinical trial comparing natural history, vitrectomy, and vitrectomy plus autologous serum: Morfields Macular Hole Study Group RAeport no. 1. Archives of Ophthalmology (Chicago, Ill : 1960) 2004, 122(2):224–236. 10.1001/archopht.122.2.224 14769600

[8] Parravano M, Giansanti F, Eandi CM, Yap YC, Rizzo S, Virgili G: Vitrectomy for idiopathic macular hole. The Cochrane Database of Systematic Reviews 2015, 2015(5):Cd009080. 10.1002/14651858.CD009080.pub2 PMC6669239 25965055

[9] Wendel RT, Patel AC, Kelly NE, Salzano TC, Wells JW, Novack GD (1993). Vitreous surgery for macular holes. Ophthalmology.

[10] Ip MS, Baker BJ, Duker JS, Reichel E, Baumal CR, Gangnon R, Puliafito CA: Anatomical outcomes of surgery for idiopathic macular hole as determined by optical coherence tomography. Archives of Ophthalmology (Chicago, Ill : 1960) 2002, 120(1):29–35. 10.1001/archopht.120.1.29 11786054

[11] Ullrich S, Haritoglou C, Gass C, Schaumberger M, Ulbig MW, Kampik A (2002). Macular hole size as a prognostic factor in macular hole surgery. Br J Ophthalmol.

[12] Wakely L, Rahman R, Stephenson J (2012). A comparison of several methods of macular hole measurement using optical coherence tomography, and their value in predicting anatomical and visual outcomes. Br J Ophthalmol.

[13] Kusuhara S, Teraoka Escaño MF, Fujii S, Nakanishi Y, Tamura Y, Nagai A, Yamamoto H, Tsukahara Y, Negi A (2004). Prediction of postoperative visual outcome based on hole configuration by optical coherence tomography in eyes with idiopathic macular holes. Am J Ophthalmol.

[14] Ruiz-Moreno JM, Staicu C, Piñero DP, Montero J, Lugo F, Amat P (2008). Optical coherence tomography predictive factors for macular hole surgery outcome. Br J Ophthalmol.

[15] Venkatesh R, Mohan A, Sinha S, Aseem A, Yadav NK (2019). Newer indices for predicting macular hole closure in idiopathic macular holes: a retrospective, comparative study. Indian J Ophthalmol.

[16] Hillenkamp J, Kraus J, Framme C, Jackson TL, Roider J, Gabel VP, Sachs HG (2007). Retreatment of full-thickness macular hole: predictive value of optical coherence tomography. Br J Ophthalmol.

[17] Oh J, Smiddy WE, Flynn HW, Gregori G, Lujan B (2010). Photoreceptor inner/outer segment defect imaging by spectral domain OCT and visual prognosis after macular hole surgery. Invest Ophthalmol Vis Sci.

[18] Chang YC, Lin WN, Chen KJ, Wu HJ, Lee CL, Chen CH, Wu KY, Wu WC: Correlation Between the Dynamic Postoperative Visual Outcome and the Restoration of Foveal Microstructures After Macular Hole Surgery. American Journal of Ophthalmology 2015, 160(1):100–106.e101. 10.1016/j.ajo.2015.03.019 25817009

[19] Chen H, Chen W, Zheng K, Peng K, Xia H, Zhu L (2015). Prediction of spontaneous closure of traumatic macular hole with spectral domain optical coherence tomography. Sci Rep.

[20] Niffenegger JH, Fong DS, Wong KL, Modjtahedi BS (2020). Treatment of secondary full-thickness macular holes with topical therapy. Ophthalmology Retina.

[21] Goto K, Iwase T, Yamamoto K, Ra E, Terasaki H (2020). Correlations between intraretinal cystoid cavities and pre- and postoperative characteristics of eyes after closure of idiopathic macular hole. Sci Rep.

[22] Sugiura Y, Okamoto F, Okamoto Y, Hiraoka T, Oshika T: RELATIONSHIP BETWEEN METAMORPHOPSIA AND INTRARETINAL CYSTS WITHIN THE FLUID CUFF AFTER SURGERY FOR IDIOPATHIC MACULAR HOLE. Retina (Philadelphia, Pa) 2017, 37(1):70–75. 10.1097/IAE.0000000000001136 27205893

[23] Kang SW, Ahn K, Ham DI (2003). Types of macular hole closure and their clinical implications. Br J Ophthalmol.

[24] Uemoto R, Yamamoto S, Aoki T, Tsukahara I, Yamamoto T, Takeuchi S (2002). Macular configuration determined by optical coherence tomography after idiopathic macular hole surgery with or without internal limiting membrane peeling. Br J Ophthalmol.

[25] Chhablani J, Khodani M, Hussein A, Bondalapati S, Rao HB, Narayanan R, Sudhalkar A (2015). Role of macular hole angle in macular hole closure. Br J Ophthalmol.

[26] Yang JM, Choi SU, Kim YJ, Kim R, Yon DK, Lee SW, Shin JI, Lee JY, Kim JG: Association between epiretinal membrane, epiretinal proliferation, and prognosis of full-thickness macular hole closure. Retina (Philadelphia*,* Pa) 2021. 10.1097/IAE.0000000000003262 34267114

[27] Yek JTO, Hunyor AP, Campbell WG, McAllister IL, Essex RW (2018). Outcomes of eyes with failed primary surgery for idiopathic macular hole. Ophthalmology Retina.

[28] Reid GA, McDonagh N, Wright DM, Yek JTO, Essex RW, Lois N: FIRST FAILED MACULAR HOLE SURGERY OR REOPENING OF A PREVIOUSLY CLOSED HOLE: Do We Gain by Reoperating?-A Systematic Review and Meta-analysis. Retina (Philadelphia, Pa) 2020, 40(1):1–15. 10.1097/IAE.0000000000002564 PMC6924931 31335482

[29] Ryan EH, Jr., Gilbert HD: Results of surgical treatment of recent-onset full-thickness idiopathic macular holes. Archives of ophthalmology (Chicago, Ill : 1960) 1994, 112(12):1545–1553. 10.1001/archopht.1994.01090240051025 7993209

[30] Willis AW, Garcia-Cosio JF (1996). Macular hole surgery. Comparison of longstanding versus recent macular holes. Ophthalmology.

