# Supplementary material for: Role of intraretinal cysts in the prediction of postoperative closure and photoreceptor damages of the idiopathic full-thickness macular hole
Source: BMC Ophthalmol. 2022 Jan 3;22:5. doi: 10.1186/s12886-021-02204-x (PMC8722226; doi:10.1186/s12886-021-02204-x)

**<Additional materials>**

**Role of intraretinal cysts in the prediction of postoperative closure and photoreceptor damages of the idiopathic full-thickness macular hole**

**Jin-Ho Joo, MD, PhD, ^1^ Woo Ho Nam, MD, ^2^ Taesung Joo, MD, ^1^ Sang Woong Moon, MD, PhD.^1^**

^1^Department of Ophthalmology, Kyung Hee University Hospital at Gangdong, Seoul, Korea

^2^Division of Ophthalmology, Department of Medicine, Kyung Hee University Graduate School.

**Supplementary Fig. 1.** Anatomica outcomes. (A) Postoperaitve type 1 closure full-thickness macular hole with intact photoreceptor. (B) Postoperaitve type 1 closure full-thickness macular hole with damaged photoreceptor. (C) Postoperaitve type 2 closure full-thickness macular hole.

**
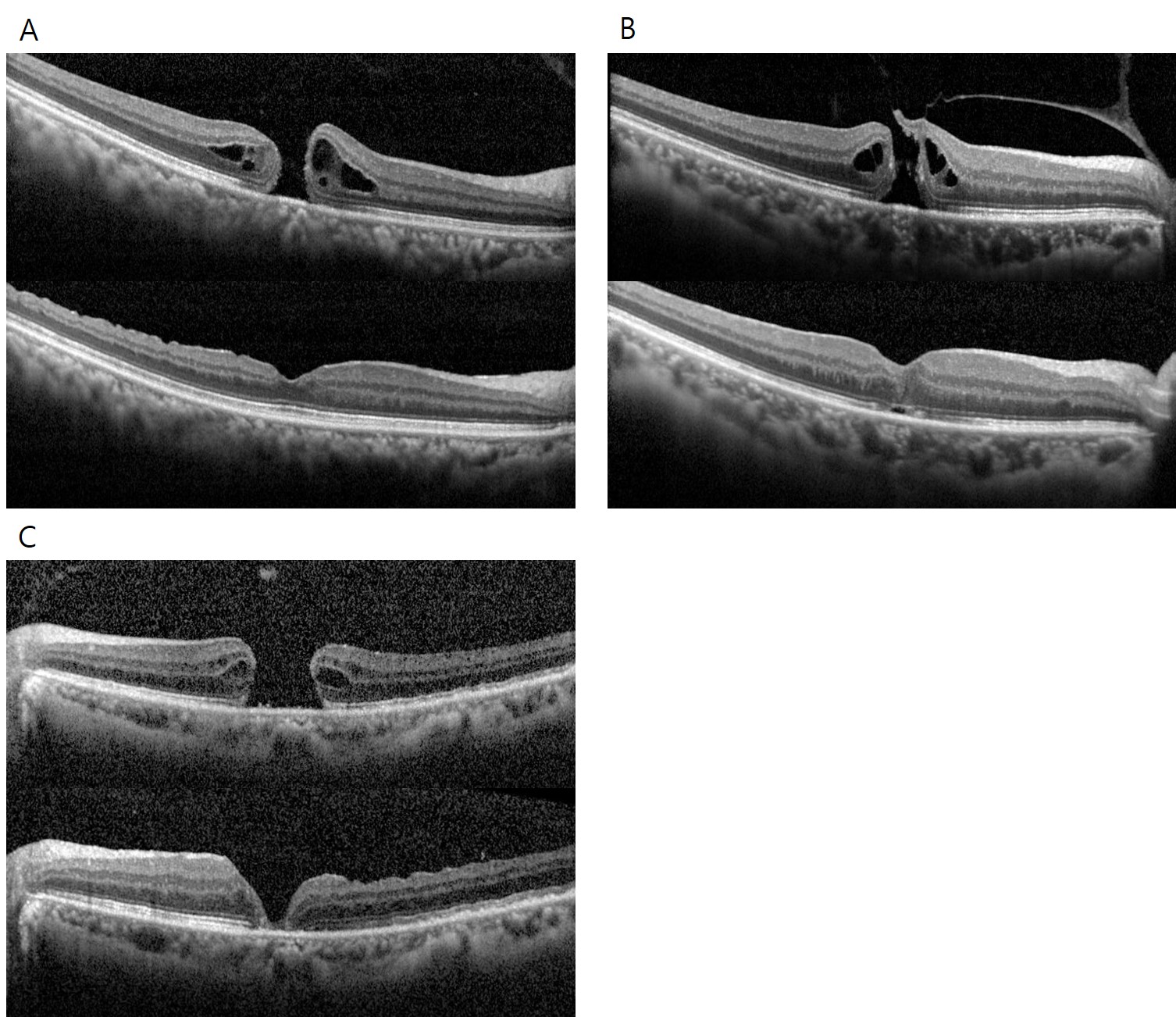
**

**Supplementary Fig. 2.** Two cases of anatomic success of full thickness macular hole (FTMH) according to presence of intraretinal cysts (IRC) after first surgical failure. Both cases had macular hole with low hole and IRC height before operation. As a result of the first operation, MH closure failed. During the postoperative follow-up, an increase in IRC height was observed, gas tamponade was performed. Finally, the FTMH was successfully closed, but an outer retinal defect remained. (A) Processed spectral domain optical coherence tomography (SD OCT) image demonstrates a small macular hole with low hole and IRC height. The measured preoperative parameters are as follows; base diameter: 459 ㎛, hole height: 318.5㎛, arm length: 141.5㎛, and IRC height: 102㎛. Postoperative 4 months SD OCT shows an increase in IRC of FTMH. The measured parameters are as follows; base diameter: 769 ㎛, hole height: 530㎛, arm length: 206㎛, IRC height: 309.5㎛. (B) Processed spectral domain optical coherence tomography (SD OCT) image demonstrates a large macular hole with low hole and IRC height. The measured preoperative parameters are as follows; base diameter: 1153 ㎛, hole height: 335.5㎛, arm length: 202.5㎛, and IRC height: 178㎛. Postoperative 1 month SD OCT shows an increase in IRC of FTMH. The measured parameters are as follows; base diameter: 1120 ㎛, hole height: 541㎛, arm length: 424㎛, IRC height: 351㎛.


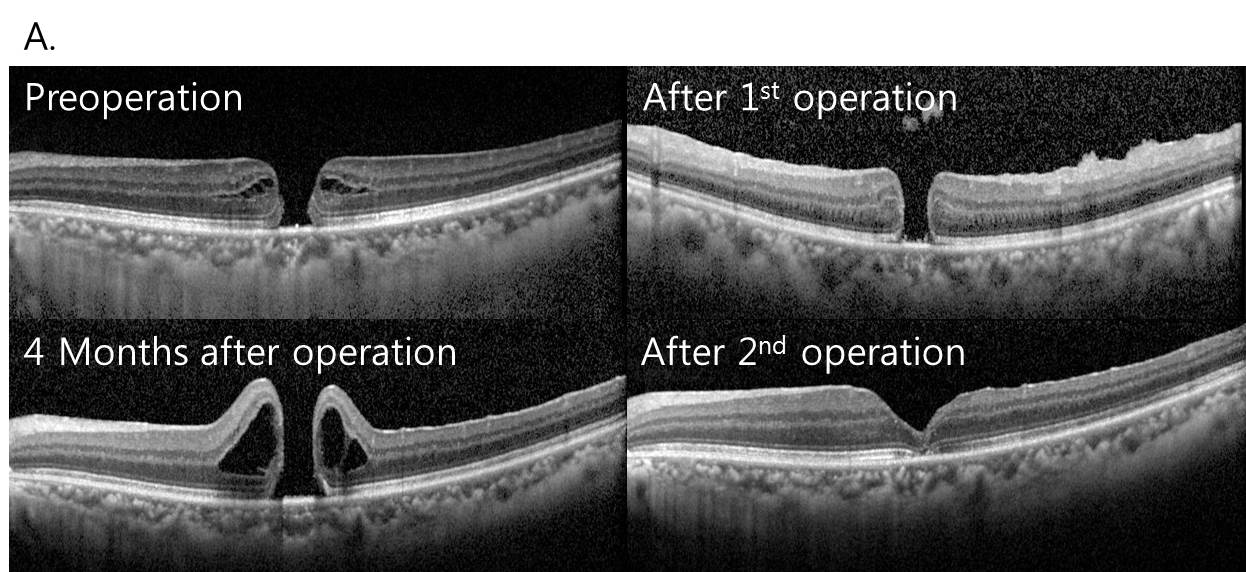


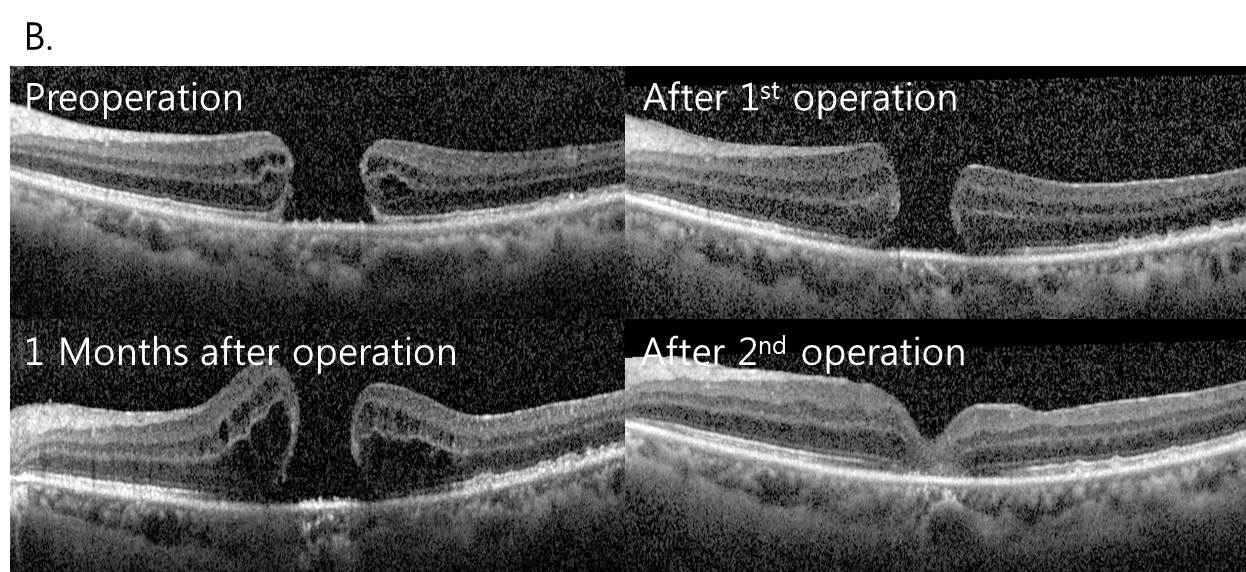

Supplement: Supplementary file 1 — Additional file 1. [file 12886_2021_2204_MOESM1_ESM.docx]
